# The Moderating Role of Community Capacity for Age-friendly Communication in Mitigating Anxiety of Older Adults During the COVID-19 Infodemic: Cross-sectional Survey

**DOI:** 10.2196/33029

**Published:** 2022-02-25

**Authors:** Frankie Ho Chun Wong, Dara Kiu Yi Leung, Edwin Lok Yan Wong, Tianyin Liu, Shiyu Lu, On Fung Chan, Gloria Hoi Yan Wong, Terry Yat Sang Lum

**Affiliations:** 1 Department of Social Work and Social Administration The University of Hong Kong Hong Kong China (Hong Kong); 2 Philip Merrill College of Journalism University of Maryland College Park, MD United States; 3 Sau Po Centre on Ageing The University of Hong Kong Hong Kong China (Hong Kong); 4 Department of Social and Behavioural Sciences City University of Hong Kong Hong Kong China (Hong Kong)

**Keywords:** COVID-19, mental health, information technology, media trust, social media, Hong Kong

## Abstract

**Background:**

Older adults were perceived as a vulnerable group during the COVID-19 pandemic due to the health and mental health challenges they faced. The pandemic was accompanied by an “infodemic” of overabundant and questionable information that has affected older adults’ mental health. As the infodemic and ageist narratives were prevalent online, more anxiety symptoms have been induced among older adults who used social media. Age-friendly communication, advocated by the World Health Organization’s Age-friendly City (AFC) guide, could be an antidote by providing tailored information via appropriate channels for older adults.

**Objective:**

This study investigated the role of community capacity for age-friendly communication in mitigating anxiety during the pandemic. We hypothesized that age-friendly communication would moderate the effects of infection risks and social media use on anxiety. A double-moderating effect was hypothesized in the context of diminished trust in traditional media.

**Methods:**

Data were collected from a cross-sectional telephone survey conducted in Hong Kong in 2020. Older adults (N=3421, age≥60 years) were interviewed about their well-being and daily lives. Community capacity for age-friendly communication was measured in a living district–based evaluation. It had 2 components: the reach of appropriate information to older adults (AFC-Information) and the age-friendliness of communication technologies (AFC-Communication Technology) in the community. We tested the hypothesized moderation and double-moderation effects with ordinary least squares regressions.

**Results:**

Perceived COVID-19 infection risk (b=0.002, *P*=.02) and use of social media for COVID-19 information (b=0.08, *P*=.04) were associated with more anxiety symptoms. The effect of using social media was moderated by AFC-Information (b=–0.39, *P*=.002) and AFC-Communication Technology (b=–1.06, *P*<.001), and the effect of perceived COVID-19 infection risk was moderated by AFC-Information (b=–0.03, *P*=.002) and AFC-Communication Technology (b=–0.05, *P*<.001). Lower trust in traditional media exacerbated anxiety symptoms associated with social media use (b=–0.08, *P*=.02). Higher AFC-Information alleviated this moderation effect (AFC-Information × media trust b=–0.65, *P*<.001; AFC-Information × social media use b=–2.18, *P*<.001; 3-way interaction b=0.40, *P*=.003).

**Conclusions:**

Our findings highlight the role of community age-friendly communication in mitigating anxiety related to the infodemic. Although using social media may have exacerbated the impact of the infodemic on older adults, it has the potential to deliver timely information for an adequate health response. Although the amplifying effects of low media trust was associated with social media use, age-friendly communication determined its strength. Instead of discouraging the use of digital technologies for COVID-19 information, efforts should be made in tailoring information and communication technologies in local communities for older adults.

## Introduction

### Background

The COVID-19 pandemic challenged older adults’ health and mental health. The threat of the pandemic may generate mental health challenges, such as anxiety, among the older population. Evidence from different countries suggests that higher COVID-19 death rates in the community are positively associated with distress in the population [1]. Another cross-national study argued that COVID-19-related anxiety is associated with the perceived vulnerability that predicts poorer well-being and increased distress [2]. Because older adults are perceived as a high-risk group, they were advised to stay at home in the early days of the pandemic. Social isolation policies, such as social distancing and lockdown, disproportionally affected the older population by heightening their risks of chronic diseases and mental health challenges [3]. A systematic review and meta-analysis estimated a 25% prevalence of COVID-19-related anxiety among the general population, where negative psychological effects can be attributed to infection risks and quarantine measures [4]. In Hong Kong, about 14% of the population showed symptoms of anxiety during the pandemic in 2020 [5], and older adults exhibited more anxiety symptoms than before the pandemic [6]. Although restrictive social isolation measures were perceived as essential to protect the older population, efforts to mitigate their anxiety were warranted.

The infodemic associated with the COVID-19 pandemic may have aggravated anxiety among older adults. Older adults obtained COVID-19-related information from more diverse sources than younger adults and were driven to worry more about the pandemic [7]. The infodemic could have engendered confusion, undermining public trust and mitigation behaviors [8]. People may panic when information from health communication is too difficult to disambiguate [9]. Conflicting information about the pandemic from media sources may also create uncertainty and stress that contribute to significant psychological issues, such as anxiety [8]. Higher anxiety levels were found among social media users during the pandemic [10,11]. COVID-19-related anxiety in older adults can be further complicated by age-related factors. Ageist views and health worries, both disproportionally affecting older adults, are associated with higher anxiety symptoms [12]. Exposure to negative-age-stereotype messaging could lead to more anxiety and less peacefulness compared to positive-age-stereotype messaging [13]. Studies on social media data suggest the pandemic was often downplayed by messages that emphasized older adults as the main population harmed by COVID-19 and their lives as less valuable [14]. The aggravating effect of the infodemic on anxiety levels can be stronger for older adults who used social media for COVID-19 information [15]. It has become essential to address the anxiety caused by social media use with age-friendly communication solutions.

Experts advocated for better media communication for older adults during the COVID-19 pandemic [16]. As in previous health crises, the public turned to the media as a crucial and reliable source of information [17]. Adequate health communication that delivers accurate information and promotes corresponding health behaviors can mitigate uncertainty and fear [18]. Specifically, effective communication of facts about communicable diseases is the key to an accurate estimation of public risks [19]. Although COVID-19 containment and public health policies may help alleviate pandemic-related mental health challenges [1,20], relevant responses should be appropriately communicated to older adults. This study investigated how the community-level capacity for age-friendly communication may help older adults navigate the pandemic and infodemic and mitigate associated anxiety.

### Community Capacity for Age-friendly Communication

According to the World Health Organization’s (WHO) guide on the Age-friendly City (AFC), “information” and “use of communication and digital devices” are 2 subdomains of age-friendly communication and information [21]. A checklist of age-friendly communication and information has been developed based on the views expressed by older people worldwide [21]. In an AFC, information of interest to older people is disseminated regularly in broadcast media and targeted media. Older people can obtain the information, orally or printed using plain language, close to their homes and where they conduct their daily activities, such as public meetings, community centers, and clubs. Volunteer callers and visitors and home support workers are some of the people who may provide information to older people who are at risk of social isolation. Regarding communication and digital devices, electronic equipment, such as mobile telephones and televisions, and automated communication are designed with age-friendly features, such as slow and clear instructions, large buttons, and big lettering. Older people can also have affordable access to computers and the internet in public places, such as community centers and libraries, with tailored instructions or individual assistance.

First, information directed to older adults at the community level may be particularly helpful in enabling them to manage the “new normal” generated by the pandemic. Complementing information on social media, information disseminated by reliable sources through familiar and preferred channels, such as telephone or information stands in the neighborhood [22], can serve as a reference for older adults when evaluating COVID-19 risk and alleviate the anxiety induced by the confusing messages on social media about the pandemic. Community information may also communicate appropriate context-specific policy responses, such as responding to local infection cases and resource distribution. Second, user-friendly features on communication and digital devices can enhance older adults’ utilization of technologies, which encourage information exchange and have the potential to remediate some of the losses they have experienced and hence maintain a vibrant and supportive community [23]. During the COVID-19 pandemic, older adults may better adapt to digital technologies designed to enhance age-friendliness to compensate for disrupted daily activities. Distributing information via communication channels with which older adults are familiar and in a timely, accessible, and affordable manner is 1 of the core AFC domains in promoting older adults’ independence and autonomy [21,24]. As a result, older adults would better mitigate the anxiety induced by COVID-19 infection risk and inconsistent misinformation from social media.

Community capacity for age-friendly communication may buffer the amplified effect of reduced trust in traditional media on infodemic-generated anxiety. In a crisis, insufficient or inconsistent information may lower public trust [25]. During the COVID-19 pandemic, the infodemic of information overabundance and misinformation has undermined public trust toward traditional institutions, including mass media, that could help deliver helpful information for older adults [26]. The prevalence of social media, where health information gains credibility by its rate of dissemination rather than scientific merit, has changed the perceived legitimacy, longstanding trust, and role of the media [27]. A cross-national study found that around 1 in 3 respondents believed the news exaggerated the pandemic [28], and evidence from a German study suggested that nearly half of respondents reported difficulty judging the trustworthiness of media information about COVID-19 [26]. Diminished media trust could undermine the effectiveness of health communication, especially compliance with protective health behaviors [29]. People who reported difficulties in ascertaining reliable guidance to cope with the pandemic exhibited mental health issues, such as anxiety [30]. Since older adults had higher risks of receiving and relaying misinformation [31], stronger efforts should be made to address the challenges they face. Community-level age-friendly communication offers a solution. A trusted information source easily accessible by older adults that can address their questions and confusion may ease their anxiety during the infodemic.

### This Study

**Figure 1:**
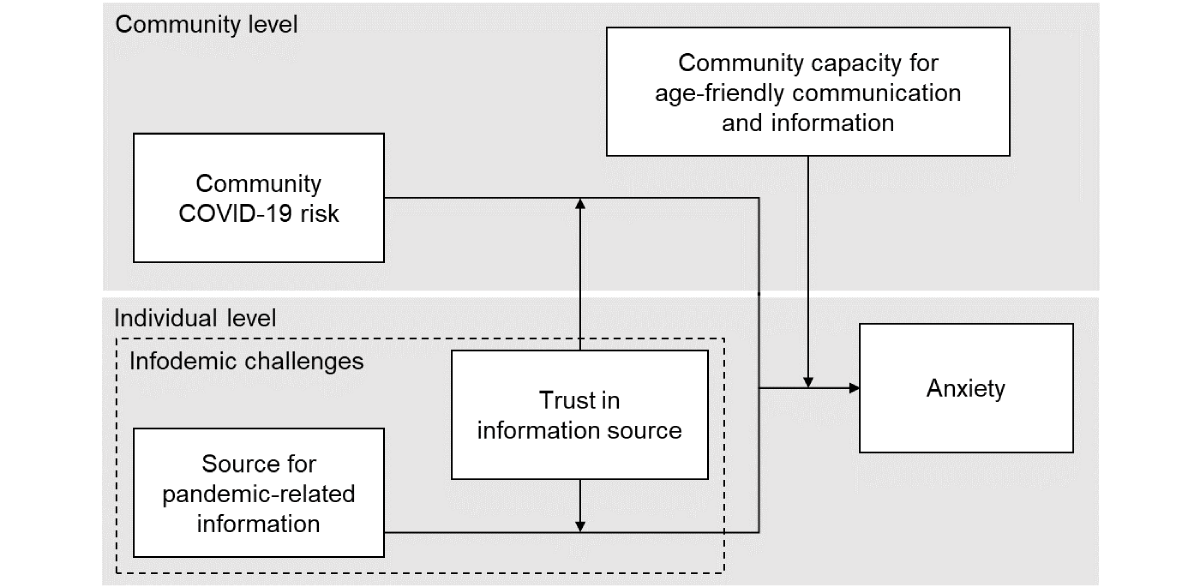
Theoretical framework.

Despite the infodemic-amplified anxiety experienced by older adults regarding public health risks during the pandemic, effective age-friendly communication on a community level could ensure they are informed and resilient against problematic information. This study investigated the factors associated with older adults’ anxiety levels and the role of age-friendly communication in moderating the effects on anxiety. We hypothesized that the risk of contracting COVID-19 and the use of social media for pandemic-related information would be associated with increased anxiety levels in older adults, where more community-level age-friendly communication could mitigate the associations. The infodemic challenged the trust in traditional media mainly by way of misinformation in social media. We hypothesized that lower trust in the media would exacerbate anxiety symptoms associated with social media use and the risk of contracting COVID-19. Nevertheless, enhancing community capacity for age-friendly communication may help alleviate the negative impact of lowered media trust. We hypothesized that higher community capacity for age-friendly communication would reduce the effect of lower trust in the media on aggravating anxiety symptoms associated with social media use and the risk of contracting COVID-19. Figure 1 illustrates the theoretical framework of this study.

## Methods

### Study Design and Sample

Respondents were recruited to answer a cross-sectional telephone survey aimed to understand the needs and well-being of community-dwelling older adults (age≥60 years) in Hong Kong during the COVID-19 pandemic. The survey protocol is described in a previous study [15]. The survey was administered between May and August 2020 to service users from community centers for older adults and community mental wellness centers. Both centers were membership based and funded by the government. Memberships are free and open to all eligible community members, which include persons aged 60 years or above for community centers for older adults and persons experiencing mental health challenges for community mental wellness centers. Community centers for older adults provide various active aging activities, such as Tai Chi, dancing, music, and computer or mobile phone classes, to their members; community mental health centers provide community health education and social support to their members. Members are eligible to enroll in various activities, usually on the first-come first-serve principle. Survey respondents were existing members of the centers, but it is unknown for how long they have been members or what types of activities they have participated in before the pandemic. The study protocol was designed by qualified clinical psychologists and researchers and pretested by frontline social workers before full-scale implementation. Trained interviewers conducted the interviews using a standardized protocol that enabled social workers to follow up respondents who exhibited mental health challenges. Of the 3550 calls made, 3421 older adults completed the interview, yielding a 96.37% success rate. No respondent had a prior COVID-19 infection history. The Human Research Ethics Committee of the University of Hong Kong approved this study (reference no. EA2003001[A]).

### Measures

#### Anxiety Symptoms

Anxiety symptoms were measured using the validated Chinese version of the 2-item Generalized Anxiety Disorder (GAD-2) questionnaire [32]. Scores range from 0 to 6, and higher scores represent more anxiety symptoms; GAD-2 score≥3 suggests the presence of an anxiety disorder [33].

#### Media Use and Trust

Respondents were asked to identify their primary source of COVID-19 information from (1) traditional media or (2) social media. The trust levels toward traditional media and social media were measured on a 5-point scale from total distrust (1) to complete trust (5). A “not applicable” option was available for each item for those who did not use the specified media type. The use of social media for COVID-19 information was captured by a 3-point ordinal scale from 0 to 2: 0, no usage (respondents selecting “not applicable” for social media trust level); 1, used social media (respondents with a valid response for social media trust level); and 2, used social media as their primary source of information.

#### Community COVID-19 Risk

The local risk of contracting COVID-19 was captured by the number of confirmed cases in the respondent’s district of residence during the week of the survey. The survey covered 12 (67%) of the 18 administrative districts in Hong Kong. This measurement was geographically sensitive and reflected the risk of contracting COVID-19 in communities in the survey period. Data were extracted from daily government reports [34].

#### Community Capacity for Age-friendly Communication

Data were extracted from assessments by the Jockey Club Age-friendly City Project in Hong Kong (Jockey Club Institute of Ageing of the Chinese University of Hong Kong et al [35]). The assessments investigated the age-friendliness of all 18 administrative districts in Hong Kong with the WHO-suggested AFC guide [21]. The same measurement method was used in previous studies in Hong Kong [36,37]. Within the communication and information domain, the subdomains “information” (AFC-Information) and “use of communication and digital devices” (AFC-Communication Technology) assessed the reach of appropriate information to older adults and the age-friendliness of communication technologies in the community, respectively. For example, the survey asked whether older adults regularly received information they found interesting and relevant to their age group and whether communication devices had large buttons and big font sizes to suit their dexterity and vision. The assessments were conducted between 2017 and 2018 by administering questionnaires with Likert scale survey questions to older adults. Average scores were obtained for each subdomain in each district. Scores ranged from 1 to 6 (1=strongly disagree to 6=strongly agree); a higher index score represented greater age-friendliness of the subdomain in the district. Although the data were obtained before the pandemic, the indexes represented the readily available capacity for age-friendly communication in communities that could be mobilized from the early stages of the pandemic.

#### Public Health Responses

Public health responses were measured by the Containment and Health Index from the Oxford COVID-19 Government Response Tracker. The index was calculated daily based on the number and strictness of containment and closure policies, such as canceling public events and stay-home requirements, and health system policies, such as contact tracing and public information campaigns [38]. Scores ranged from 0 to 100; a higher index score indicated that more containment measures were in place. The index score of the interview date was aligned to each respondent to control for its effects on anxiety levels.

#### Demographic Covariates

Demographics collected included age in years, gender (0=male, 1=female), district of residence, and service nature. District of residence was not included in the main analysis but was used to match respondents’ community COVID-19 risk and AFC indexes. Service nature was indicated by respondents’ involvement with either community aged care services or a mental wellness center.

### Statistical Analysis

Descriptive statistics were computed and appropriately reported. All hypotheses were tested by hierarchical ordinary least squares (OLS) regressions. All models were controlled by the Containment and Health Index and demographic covariates. First, baseline models predicting the GAD-2 score were estimated. Independent variables in the baseline model included community COVID-19 risk and social media use. Since the 2 moderator variables, AFC-Information and AFC-Communication Technology indexes, were substantially correlated (r=0.48, *P*<.001), they were included in 2 baseline models separately. The variance inflation factors of all variables in all baseline models were below 2.0, suggesting low multicollinearity between the variables. Second, 4 OLS regression models examined the mediation effects between the independent variables and moderators. The final set tested for moderation and double-moderation effects with trust in traditional media. Graphs of predicted values are provided to illustrate the moderation effects. The Johnson-Neyman technique was used to identify the range of significant moderation effects [39]. Sensitivity analyses using binary independent variables as social media use measurements, log-transformed GAD-2 score as a dependent variable in OLS regression models, Poisson regressions, and 2-part mixed models yielded similar results. The current set of OLS models is presented for better comprehension and interpretation. Statistical analysis was conducted using R.

## Results

### Demographics

Table 1 shows respondents’ (N=3421) demographic characteristics. Their average age was 76 years (SD 8.9), 2549 (74.58%) of 3418 respondents were female, and 2666 (77.93%) of 3421 respondents were members of community centers for older adults. The average Containment and Health Index score was 58.57 (SD 8.80) within the 119-day interview time frame, and there were on average 25.7 (SD 27.5) COVID-19 cases within communities when the survey was conducted. The average GAD-2 score was 0.74 (SD 1.2), where 239 (7.0%) of the 3421 respondents were at risk of anxiety (GAD-2 score≥3), suggesting anxiety symptoms were not prevalent among respondents. Trust in traditional media was moderately high and averaged 4.27 (SD 0.88). Around 1399 (40.89%) of the 3421 respondents used social media and rated their trust in social media, and the average score was 3.18 (SD 1.1); in addition, 203 (5.93%) indicated that social media was their main source of COVID-19 information. The average AFC-Information index was 4.09 (SD 0.21) and the AFC-Communication Technology index 3.96 (SD 0.13).

**Table 1:** Respondents’ characteristics (N=3421).

Variables		Total respondents, N	Respondents, n (%)	Mean (SD)	
**Demographics**					
	Age (years)	3421	—^a^	76 (8.9)	
	Gender (female)	3418	2549 (74.58)	—	
	Service nature (community center for older adults)	3421	2666 (77.93)	—	
	Containment Health Index (range 0-100, 119 days)	3421	—	58.5 (8.8)	
**Psychological distress**					
	GAD-2^b^ score (range 0-6)	3388	—	0.74 (1.2)	
**Community COVID-19 risk**					
	Weekly number of COVID-19 cases in district (range 0-135)	3421	—	25.7 (27.5)	
**Trust in traditional media (range 1-5)**		3335	—	4.27 (0.88)	
**Trust in social media (range 1-5)**		1399	—	3.18 (1.1)	
**Using social media for COVID-19 information**					
	Used social media for COVID-19 information	3421	1399 (40.89)	—	
	Social media as the main source of COVID-19 information	3421	203 (5.93)	—	
**Community capacity for age-friendly communication (12 districts)**					
	AFC^c^-Information index (range 1-6)	3421	—	4.09 (0.21)	
	AFC-Communication Technology index (range 1-6)	3421	—	3.96 (0.13)	

^a^Not applicable.

^b^GAD-2: 2-item Generalized Anxiety Disorder.

^c^AFC: Age-friendly City.

Table 2 and Table 3 present the associations between anxiety symptoms and the independent variables by OLS regressions. The baseline model shows that the GAD-2 score was positively associated with higher community COVID-19 risk (b=0.002, *P*=.02) and social media use (b=0.08, *P*=.04). A higher Containment and Health Index score, meanwhile, was negatively associated with the GAD-2 score (b=–0.02, *P*<.001). Female respondents exhibited more anxiety symptoms (b=0.22, *P*<.001), but those who received service from a community center for older adults showed less anxiety symptoms (b=–0.55, *P*=.001). The baseline models with AFC indexes suggested a positive association between anxiety symptoms and AFC-Information (b=0.23, *P*=.002) and AFC-Communication Technology (b=0.99, *P*<.001).

**Table 2:** OLS^a^ regression results predicting anxiety level moderated by the AFC^b^-Information index (N=3385).

Variables	Baseline			With AFC-Information			Social media use × AFC-Information			COVID-19 risk × AFC-Information	
	b	*P* value	b		*P* value	b		*P* value	b		*P* value
Age	–0.002	.39	–0.001		.44	–0.003		.33	–0.004		.11
Gender (female)	0.22	<.001	0.22		<.001	0.22		<.001	0.22		<.001
Service nature (aged care)	–0.55	<.001	–0.58		<.001	–0.59		<.001	–0.60		<.001
Containment Health Index	–0.02	<.001	–0.02		<.001	–0.02		<.001	–0.03		<.001
Community COVID-19 risk	0.002	.02	0.001		.07	0.001		.05	0.14		<.001
Social media use	0.08	.04	0.08		.03	1.71		.001	0.08		.03
AFC-Information	—^c^	—	0.23		.002	0.42		<.001	1.10		<.001
Social media use × AFC-Information	—	—	—		—	–0.39		.002	—		—
COVID-19 risk × AFC-Information	—	—	—		—	—		—	–0.03		<.001
Adjusted R^2^	0.066	—	0.069		—	0.071		—	0.084		—

^a^OLS: ordinary least squares.

^b^AFC: Age-friendly City.

^c^Not applicable.

**Table 3:** OLS^a^ regression results predicting anxiety level moderated by the AFC^b^-Communication Technology index (N=3385).

Variables	Baseline			With AFC-Communication Technology			Social media use × AFC-Communication Technology			COVID-19 risk × AFC-Communication Technology	
	b	*P* value	b		*P* value	b		*P* value	b		*P* value
Age	–0.002	.39	–0.002		.33	–0.003		.19	–0.004		.13
Gender (female)	0.22	<.001	0.21		<.001	0.22		<.001	0.21		<.001
Service nature (aged care)	–0.55	<.001	–0.63		<.001	–0.62		<.001	–0.66		<.001
Containment Health Index	–0.02	<.001	–0.03		<.001	–0.03		<.001	–0.03		<.001
Community COVID-19 risk	0.002	.02	0.001		.10	0.001		.18	0.20		<.001
Social media use	0.08	.04	0.07		.06	4.31		<.001	0.05		.16
AFC-Communication Technology	—^c^	—	0.99		<.001	1.50		<.001	1.81		<.001
Social media use × AFC-Communication Technology	—	—	—		—	–1.06		<.001	—		—
COVID-19 risk × AFC-Communication Technology	—	—	—		—	—		—	–0.05		<.001
Adjusted R^2^	0.066	—	0.076		—	0.081		—	0.088		—

^a^OLS: ordinary least squares.

^b^AFC: Age-friendly City.

^c^Not applicable.

**Figure 2:**
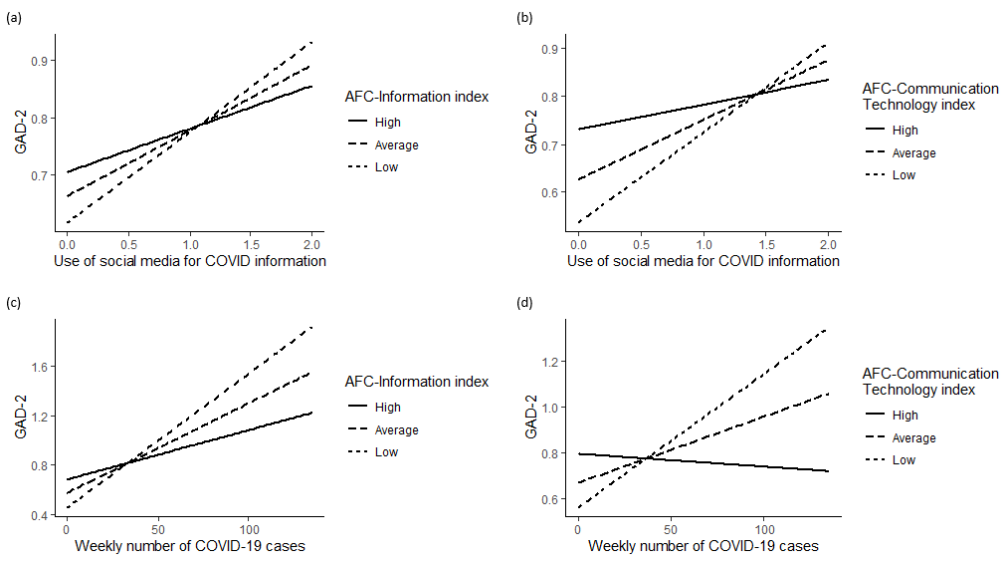
Moderation effects of AFC-Information and AFC-Communication Technology indexes. AFC: Age-friendly City; GAD-2: 2-item Generalized Anxiety Disorder.

Nevertheless, the effects of COVID-19 risk and social media use on anxiety level depended on the age-friendliness of community communication. OLS models with interaction terms suggested significant moderation effects by the AFC-Information index and the AFC-Communication Technology index. The effect of social media use on anxiety symptoms was moderated by AFC-Information (b=–0.39, *P*=.002) and AFC-Communication Technology (b=–1.06, *P*<.001). Figures 2a and 2b illustrate the moderated relationships. The ranges of significant moderation slopes suggested by the Johnson-Neyman technique were AFC-Information<4.20 and AFC-Communication Technology<4.01. The predicted anxiety symptoms of older adults living in a community with high AFC-Information and AFC-Communication Technology indexes were not associated with social media use. More social media use predicted higher GAD-2 scores among older adults living in a community with AFC indexes lower than the thresholds, and the associations were stronger in communities with lower AFC indexes. The effect of community COVID-19 risk was also moderated by AFC-Information (b=–0.03, *P*<.001) and AFC-Communication Technology (b=–0.05, *P*<.001). Figures 2c and 2d illustrate the moderated relationships. Significant ranges of slopes were AFC-Information<4.27 or AFC-Information>4.36 and AFC-Communication Technology<3.99 or AFC-Communication Technology>4.05. Community COVID-19 risk was positively associated with predicted GAD-2 scores in communities with AFC indexes lower than the thresholds, whereas the associations were negative in communities with AFC indexes higher than the thresholds. In general, in districts with a lower capacity for communicating with older adults, more social media use and higher community COVID-19 risk were associated with more anxiety symptoms.

When trust in traditional media was considered in the moderated relationships, the 3-way-interaction OLS regression models suggested that AFC-Information is the key to moderating the effects on GAD-2 scores but not AFC-Communication Technology. Table 4 presents the moderation effects and the double-moderation effects with trust in traditional media. The single-moderation model suggested trust in traditional media moderates the effects of social media use (b=–0.08, *P*=.02) but not community COVID-19 risk (b=–0.000, *P*=.77). The significant moderation slope range suggested by the Johnson-Neyman technique was trust in traditional media<3.93. For older adults with lower trust in traditional media, using more social media predicted more anxiety symptoms. The double-moderation models are consistent with previous findings. In addition, trust in traditional media and AFC-Information exhibited a double-moderation effect with social media use (3-way interaction b=0.40, *P*=.003) and community COVID-19 risk (3-way interaction b=0.01, *P*=.01). Meanwhile, trust in traditional media and AFC-Communication Technology showed no significant double-moderating effect with social media use (3-way interaction b=0.35, *P*=.14) and community COVID-19 risk (3-way interaction b=–0.004, *P*=.62). Table 5 summarizes the 3-way-interaction effects between AFC-Information, trust for traditional media, social media use, and the weekly number of COVID-19 cases on anxiety.

**Table 4:** OLS^a^ regression results predicting anxiety level, 3-way interaction (N=3300).

Variables	Baseline			Social media use × media trust			COVID-19 risk × media trust			AFC^b^-Information double moderation			AFC-Communication Technology double moderation	
	b	*P* value	b		*P* value	b		*P* value	b		*P* value	b		*P* value
Age	–0.002	.49	–0.002		.43	–0.002		.49	–0.004		.08	–0.005		.08
Gender (female)	0.20	<.001	0.20		<.001	0.20		<.001	0.20		<.001	0.20		<.001
Service nature (aged care)	–0.54	<.001	–0.54		<.001	–0.54		<.001	–0.60		<.001	–0.63		<.001
Containment Health Index	–0.02	<.001	–0.02		<.001	–0.02		<.001	–0.02		<.001	–0.02		<.001
Community COVID-19 risk	0.002	.02	0.002		.02	0.003		.46	0.38		<.001	0.12		.40
Social media use for COVID-19 information	0.06	.11	0.40		.01	0.06		.11	9.4		<.001	10.4		.01
Traditional media trust	–0.04	.08	–0.002		.96	–0.04		.24	2.7		<.001	0.73		.46
Social media use × media trust	—^c^	—	–0.08		.02	—		—	–1.7		.002	–1.5		.12
COVID-19 risk × media trust	—	—	—		—	–0.000		.77	–0.06		.01	0.02		.64
AFC-Information	—	—	—		—	—		—	4.0		<.001	—		—
Social media use × AFC-Information	—	—	—		—	—		—	–2.2		<.001	—		—
COVID-19 risk × AFC-Information	—	—	—		—	—		—	–0.09		<.001	—		—
Social media use × media trust × AFC-Information	—	—	—		—	—		—	0.40		.003	—		—
COVID-19 risk × media trust × AFC-Information	—	—	—		—	—		—	0.01		.01	—		—
AFC-Communication Technology	—	—	—		—	—		—	—		—	3.0		.004
Social media use × AFC-Communication Technology	—	—	—		—	—		—	—		—	–2.5		.01
COVID-19 risk × AFC-Communication Technology	—	—	—		—	—		—	—		—	–0.03		.42
Social media use × media trust × AFC-Communication Technology	—	—	—		—	—		—	—		—	0.35		.14
COVID-19 risk × media trust × AFC-Communication Technology	—	—	—		—	—		—	—		—	–0.004		.62
Adjusted R^2^	0.065	—	0.066		—	0.065		—	0.092		—	0.092		—

^a^OLS: ordinary least squares.

^b^AFC: Age-friendly City.

^c^Not applicable.

**Table 5:** Summary of the 3-way interaction on anxiety level.

AFC^a^-Information index	Media trust	Association between social media use for COVID-19 information and anxiety	Association between weekly number of COVID-19 cases and anxiety
Low	High	Insignificant	Weaker
Low	Low	Stronger	Stronger
High	High	Insignificant	Insignificant
High	Low	Insignificant	Insignificant

^a^AFC: Age-friendly City.

**Figure 3:**
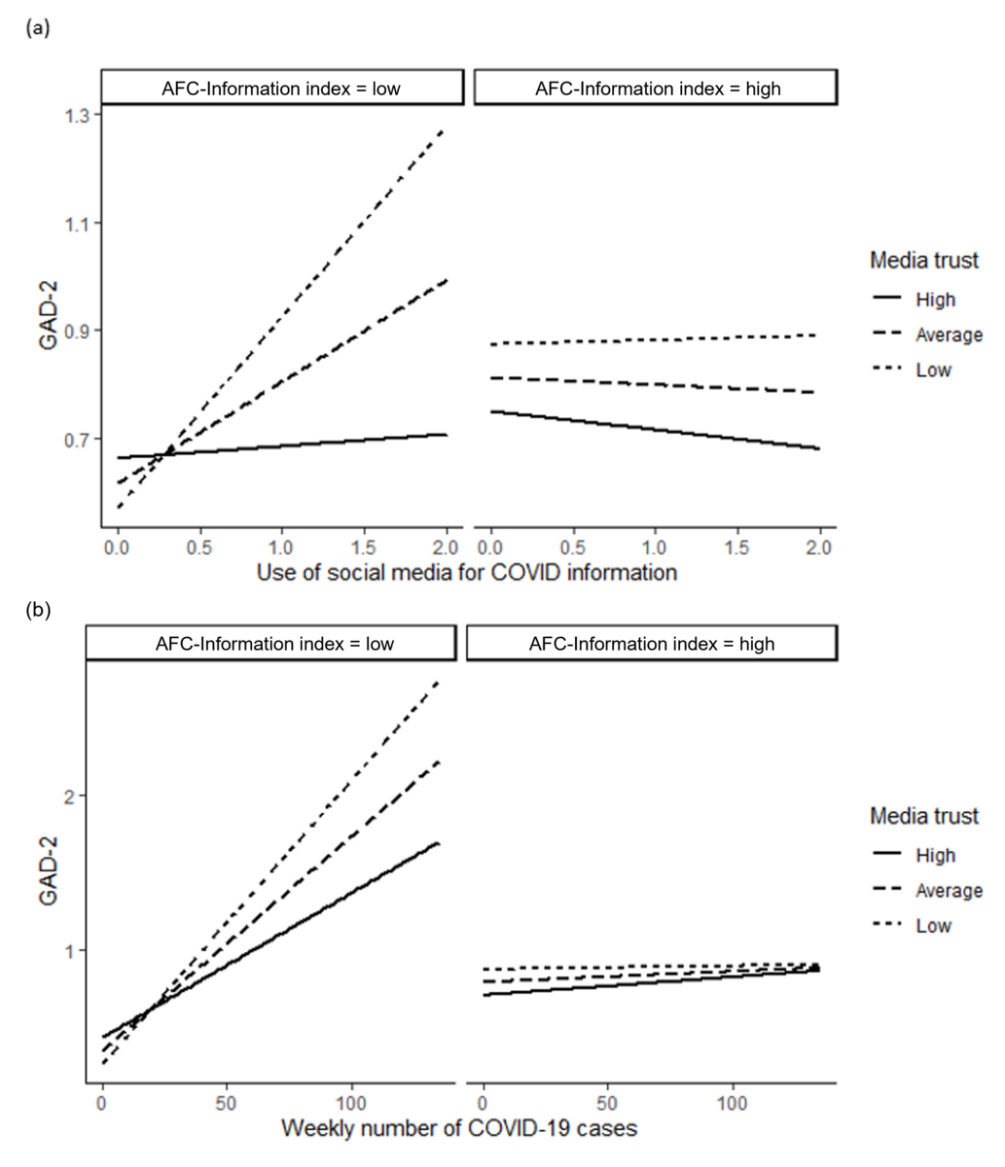
Double-moderation effect of the AFC-Information index. AFC: Age-friendly City; GAD-2: 2-item Generalized Anxiety Disorder.

Figure 3a illustrates the double-moderation effect of trust in traditional media and AFC-Information with social media use on GAD-2 scores. Media trust significantly moderated the effect of social media use on anxiety symptoms for older adults living in low-AFC-Information communities. For example, when AFC-Information was 1 SD below the mean (AFC-Information=3.88), more social media use significantly predicted more anxiety symptoms if media trust was lower. The Johnson-Neyman technique revealed that the slope of moderation remained significant when media trust<4.63. However, when AFC-Information was 1 SD above the mean (AFC-Information=4.30), trust in traditional media no longer significantly moderated the effect of social media use on anxiety symptoms. Figure 3b illustrates the double-moderation effect of trust in traditional media and AFC-Information with community COVID-19 risk on GAD-2 scores. Media trust moderated the effect of community COVID-19 risk on GAD-2 scores for older adults living in low-AFC-Information communities. When AFC-Information was 1 SD below the mean (AFC-Information=3.88), a higher community COVID-19 risk predicted more anxiety symptoms if media trust was lower. Results from the Johnson-Neyman technique suggest the moderation effect was significant for media trust<5.64. Similarly, when AFC-Information was higher, the moderation effect of media trust became insignificant. If AFC-Information was 1 SD above the mean (AFC-Information=4.30), trust in traditional media showed no moderation effect on the relationship between community COVID-19 risk and GAD-2 scores. In summary, higher AFC-Information alleviated the anxiety generated by social media use and higher community COVID-19 risk that was associated with low trust in traditional media.

## Discussion

### Principal Findings

Study findings suggest that age-friendly communication offers community-level protection on mental health in an unprecedented crisis such as the COVID-19 pandemic by moderating the anxiety associated with COVID-19 infection risk. Although a higher COVID-19 infection risk may generate perceived vulnerability [2], this study found that adequate information communicated with older adults may alleviate the anxiety associated with community COVID-19 risk. When older adults obtain a better picture of the developments of the pandemic and corresponding coping strategies, their anxiety about the potential health threats may diminish. In the meantime, results show that older adults living in districts with more age-friendly communication and digital devices experience less anxiety associated with perceived COVID-19 infection risk. Technology usage may be associated with older adults’ coping strategies during times of reduced social contact. When community COVID-19 risk increases, older adults with access to information and communication technology devices could supplement or substitute their daily activities in the community with online alternatives [40]. They could stay connected with family members and use teleconferencing to access social and medical services [40,41]. As a result, the flexibility of these older adults allowed them to engage in daily activities in the “new normal.” The anxiety associated with increased infection risk was moderated.

This study found that the anxiety associated with social media use for COVID-19 information is moderated by age-friendly communication. Although higher social media usage for COVID-19 information was positively associated with anxiety symptoms among older adults, community capacity for age-friendly communication may moderate the association. First, the perception of being better informed may lower pandemic-related anxiety [42]. Information that was adapted to reach older adults could provide an anchor point for those using social media and encountering inconsistent and incorrect information online. Community-level information valued by older adults typically originated from the public and voluntary sectors, which have strong roles in providing directed information through telephone and bulletin boards in key locations [21]. When the information sources are institutions in which older adults have developed trusting relationships over time, the communication process occurs proximally and addresses the unique context surrounding their neighborhoods. Older adults could reference information from the community to evaluate the health risks they were exposed to even under the infodemic. Moreover, communication technologies that are designed for older adults may provide a smoother user experience. Although low levels of comfort and control over technologies and cognitive challenges among older adults are considered causes of anxiety [43], age-friendly technologies in the community may help them better navigate the digital environment with reduced anxiety.

A key contribution of this study is the demonstration of the double-moderating effect of community-based information for older adults on anxiety during the infodemic. On the one hand, our results suggest that older adults who have lower trust in the media show more anxiety symptoms when they use more social media for COVID-19 information. Distrust in mainstream information may have hindered older adults’ ability to judge the quality of information appropriately. Problematic information thus could impose a stronger anxiety-inducing effect [8,9]. On the other hand, our analysis found a double-moderation effect of age-friendly community information on anxiety. Existing studies have focused on the association between anxiety and information consumption behavior at the individual level, such as using social media [10,11,15]. This study expanded the examination to the information provision at the community level and the interplay between the individual and community levels. Although lower media trust may amplify the effects of social media use and community COVID-19 risk on anxiety, information available in the community for older adults determines the strength of these associations. In other words, community information mitigates the negative effects of low media trust. Even when the infodemic undermined media trust, older adults were less likely to exhibit associated anxiety symptoms.

There are several possible explanations for our findings. First, information from the community possibly overshadowed other information, diluting the effects of media trust on inducing anxiety. Studies on media use suggest that news consumption can be a ritualized and habitual behavior [44]. Therefore, when older adults are able to obtain relevant information about the pandemic and coping strategies from their routinely used information source in the community, they may pay less attention to the media for answers to resolve their concerns. It could reduce the effect of media mistrust. Second, information from the community may have served as a strong reference for older adults to determine the trustworthiness of questionable information they encountered. Since community information was mainly circulated by trusted parties outside the media [21], older adults may juxtapose it with online information to obtain a reliable judgment. Third, age-friendly communication retains the crucial element of societal engagement by providing a “gathering place” for older adults to stay connected with their community [45,46]. Anxiety induced by the infodemic on social media and distrust toward information from media sources may be mitigated by information from the community via informal interpersonal communication. Older adults value not only the clarifications obtained in conversations but also the attention from a real person [21]. Communication sustained in the community may provide the buffer for the problematic information that older adults receive online, especially when they have lower trust in the media.

This study provides evidential support for advocating age-friendly communication in local communities. On technology usage, although information delivered offline through key locations and persons is easily accessible for most older adults [22], appropriately used digital technologies may further strengthen the communication process [23]. The digital divide should be handled carefully to ensure that older adults facing the double burden of social and digital exclusion can receive support to use technology for communication and information purposes in the pandemic [47]. Providing age-friendly devices alone is not sufficient—community resources should be directed at peer learning opportunities and translating technical language to age-friendly instructions for establishing digital skills effectively [45]. More importantly, solutions should be context specific and capable of addressing challenges faced in the community [48]. In any event, media literacy education should be provided to older adults to enable community-based communication to serve as a crucial channel for promoting information consumption and critical evaluation of health information. Essential skills to debunk myths and clarification of the latest misinformation can be circulated timely at the local level. Considering the increasing use of social media for health information among older people and its relation to their heightened anxiety in a health crisis [10,11,15], the AFC framework on communication and information can be expanded to include the community’s general age-friendly capacity for and utilization of social media communication, moving beyond the current scope of specifically examining the instructions provided to the population on operating digital devices. Furthermore, the interpersonal network upheld by community-level communication may help consolidate the wisdom between older adults. Resilience can be built where older adults may seek answers from peers and community partners when the pandemic threat is heightened or when they encounter questionable information via the infodemic.

### Limitations

The cross-sectional nature of this study means that identified associations should not be treated as causal relationships. The questionnaire was designed to be brief to facilitate expedited completion and extensive reach to older adults. Therefore, the instruments were chosen for their conciseness but provided limited detailed information. For example, social media use for COVID-19 information was constructed as a 3-point measurement. This may have lowered its sensitivity to detect actual usage frequency and hence the estimated effects of social media usage. Data quality could have been affected by the self-reported nature of the survey in terms of memory loss and social desirability bias. Since the survey respondents had established relationships with social services, which may have contributed to the high response rate, they could have utilized more community resources and had higher trust toward the information shared in the community than the general older population. Meanwhile, since the age-friendly communication variables were obtained before the COVID-19 pandemic, community capacity for age-friendly communication could have changed when older adults were later interviewed during the disruption of social life. Age-friendly social services and community resources may be inaccessible for the time being, and hence older adults would not have benefited from them. Although this study suggests the moderation effect of community capacity for age-friendly communication, the measurements did not cover the actual usage of relevant resources. Our findings may underestimate the effect for older adults who fully utilized age-friendly communication opportunities. Lastly, trust in traditional media alone was measured to gauge the influence of the infodemic. This measurement may not fully reflect the impact of the infodemic, and more dimensions of the infodemic are still worth investigating.

### Conclusion

Although perceived infection risks and social media use during the COVID-19 pandemic may induce anxiety among older adults, community capacity for age-friendly communication alleviates their effects. By lowering trust in traditional media, the infodemic may amplify the effects of perceived infection risks and social media use on anxiety. However, better information circulation in the community for older adults moderates the influence of low media trust. Context-specific age-friendly communication solutions can mitigate the anxiety intensified by the infodemic. Although it is important to curate and deliver age-specific information for older adults, efforts should be made to build older adults' capacity in evaluating and sharing useful information amid the infodemic.

## Abbreviations

AFC: Age-friendly City
GAD-2: 2-item Generalized Anxiety Disorder
OLS: ordinary least squares
WHO: World Health Organization
